# Preoperative upper tract invasive diagnostic modalities are associated with intravesical recurrence following surgery for upper tract urothelial carcinoma: A population-based study

**DOI:** 10.1371/journal.pone.0281304

**Published:** 2023-02-02

**Authors:** Fredrik Liedberg, Oskar Hagberg, Christel Häggström, Firas Aljabery, Truls Gårdmark, Abolfazl Hosseini, Staffan Jahnson, Tomas Jerlström, Viveka Ströck, Karin Söderkvist, Anders Ullén, Lars Holmberg, Johannes Bobjer

**Affiliations:** 1 Department of Urology Skåne University Hospital, Malmö, Sweden; 2 Institution of Translational Medicine, Lund University, Malmö, Sweden; 3 Department of Surgical Sciences, Uppsala University, Uppsala, Sweden; 4 Department of Public Health and Clinical Medicine, Northern Register Centre, Umeå University, Umeå, Sweden; 5 Division of Urology, Department of Clinical and Experimental Medicine, Linköping University, Linköping, Sweden; 6 Department of Clinical Sciences, Danderyd Hospital, Karolinska Institute, Stockholm, Sweden; 7 Department of Molecular Medicine and Surgery, Karolinska Institutet, Stockholm, Sweden; 8 Department of Urology, School of Medical Sciences, Faculty of Medicine and Health, Örebro University, Örebro, Sweden; 9 Department of Urology, Sahlgrenska University Hospital and Institute of Clinical Sciences, Sahlgrenska Academy, University of Gothenburg, Gothenburg, Sweden; 10 Department of Radiation Sciences, Umeå University, Umeå, Sweden; 11 Department of Oncology-Pathology, Karolinska Institutet, Stockholm, Sweden; 12 Department of Pelvic Cancer, Genitourinary Oncology and Urology Unit, Karolinska University Hospital, Stockholm, Sweden; 13 School of Cancer and Pharmaceutical Sciences, King’s College London, London, United Kingdom; IRCCS Giovanni Paolo II Cancer Hospital, ITALY

## Abstract

**Background:**

Intravesical recurrence (IVR) after surgery for upper tract urothelial carcinoma (UTUC) is a clinical problem. We investigated if preoperative invasive diagnostic modalities (IDM) such as antegrade/retrograde uretero-pyelography and/or selective urine cytology/barbotage, and URS with or without concomitant biopsy are associated with IVR after radical surgery for UTUC. Risk of death from urothelial cancer and all causes was investigated as secondary outcomes.

**Methods:**

We investigated a population-based cohort of 1038 consecutive patients subjected to radical surgery for UTUC 2015–2019 in Sweden, using the Bladder Cancer Data Base Sweden (BladderBaSe 2.0), comprising all patients in the Swedish National Registry of Urinary Bladder Cancer. Risk estimates of IVR, death from urothelial cancer, and all causes was assessed using multivariable Cox regression models.

**Results:**

The study included 536 cases with and 502 without preoperative IDM. IDM was associated with increased risk of IVR (HR 1.24, 95% CI 1.03–1.52) and risk of urothelial cancer death (HR 1.56, CI 1.12–2.18), compared to no IDM after a median follow-up of 1.3 yrs. Stratified analysis for tumor location showed that IDM was associated with risk of IVR in ureteric cancer (HR 1.66, 95% CI 1.21–2.28) but not in renal pelvic cancer (HR 1.07, 95% CI 0.81–1.41). Limitations included the observational setting and the lack of variables such as tumour grade, multifocality and preoperative hydronephrosis.

**Conclusions:**

Worse outcomes for patients subjected to preoperative IDM highlight the need for carefully considering diagnostic decisions for UTUC patients, specifically in tumours located in the ureter.

## Introduction

Optimal diagnostic accuracy and/or risk stratification in upper tract urothelial carcinoma (UTUC) sometimes require invasive diagnostic modalities (IDM) such as ureteroscopy (URS) with or without biopsy [1]. However, such diagnostic procedures may increase risk of tumour seeding and future intravesical recurrence (IVR) [2]. By similar mechanisms, other preoperative diagnostic procedures such as antegrade/retrograde uretero-pyelography or collection of selective urine cytology/barbotage may also imply increased risk of IVR [3]. Existing data, mainly based on small retrospective series which also have been reviewed systematically and pooled in meta-analyses [2, 4] as well as one larger single-centre cohort [5] indicate an increased risk of IVR for patients subjected to preoperative diagnostic URS. In contrary, in the largest study to date based on a Taiwanese population-based dataset no such increased risk was detected [6].

Recent advancements in perioperative systemic therapy strategies in UTUC [7, 8] further highlight the importance of a thorough diagnostic workup, which includes choosing the best timing for systemic therapy in light of potential lower kidney function following surgery [9]. Possibly, further understanding of UTUC molecular variants might affect both diagnostic and treatment strategies [10].

Our primary study aim was to assess the risk of IVR in a population-based nation-wide cohort also with evaluation of other IDM in addition to URS. Thus, we analysed all patients treated with extirpative surgery (radical nephroureterectomy (RNU), segmental ureteric resection, or nephrectomy only) between years 2015–2019 in the Bladder Cancer Data Base Sweden (BladderBaSe 2.0), comprising all patients in the Swedish National Registry of Urinary Bladder Cancer (SNRUBC) with registered information on diagnostic modalities including any instrumentation of the upper urinary tract used as exposure. As secondary end-point, we also assessed the association between IDM and survival.

## Patients and methods

### Study population

We identified 1094 UTUC-patients treated with RNU, segmental ureteric resection, or nephrectomy only from the start of registration of patients with UTUC in January 2015 until mid 2019 (distribution per year is available in the S1 Table) in the BladderBaSe 2.0 [11]. We excluded patients with primary metastatic disease registered at diagnosis (stage M1) (n = 56), leaving 1038 patients eligible for analysis.

### Measures

Individual patient data regarding treatment and individual diagnostic modalities were retrieved from the SNRUBC.

Standard monitoring of the bladder to detect IVR during follow-up after RNU according to the Swedish national guidelines include cystoscopy at 4 and 12 months and then annually until 5 years for unifocal low grade tumours <2 cm, with an additional cystoscopy at 8 months for high risk tumours [12]. Additionally, follow up for high risk tumours include voided urine cytology at every cystoscopy, as well as imaging to detect metastases and metachronous UTUC.

IVR was defined as a bladder cancer diagnosis registration after date of UTUC surgery in the SNRUBC. For patients with previous bladder cancer (n = 287), IVR was defined as a registration of a transurethral resection (TUR-B, KCD02), cystoscopy with a biopsy (UKC05), or radical cystectomy (KCC) in linked data from the National in- and outpatient registries after the date of UTUC surgery.

Risk of death from urothelial cancer and other causes were assessed by merged data from the Swedish cause of death registry. Death from urothelial cancer (cancer-specific death) was defined as urothelial cancer in the kidney pelvis (ICD-10 code C65), ureter (C66), bladder (C67) or urethra (C68) as underlying death causes.

Charlson Comorbidity Index (CCI) [13] was calculated based on a list of diseases, with a specific weight assigned to each disease category according to data from the National patient register. The separate weights were collated to give an overall score, categorising morbidity as follows: 0 = none, 1 = mild, 2 = intermediate, and ≥ 3 = severe. Educational level data was retrieved from Statistics Sweden and categorised as low (≤ 9 years of education), intermediate (10–12 years), and high (≥ 13 years), corresponding to mandatory school, high school, and college or university [11].

### Statistical analyses

Patients were stratified in groups based on increasingly invasive diagnostic modalities (IDM) as follows: In addition to cystoscopy and a computed tomography (CT) urography or a magnetic resonance tomography (MRT), either A) voided urine cytology, B) retrograde/antegrade pyelography and/or selective urine cytology, C) ureteropyeloscopy with or without barbotage for cytology, or D) ureteropyeloscopy with tumour biopsy. Patients were further categorised as IDM- (A) or IDM+ (B, C and D) for further comparison of patients with or without preoperative instrumentation of the upper urinary tract. If patients matched several criteria they were categorized according to the most invasive modality (e.g. one patient with both A and B was given B in the calculations).

The Kaplan-Meier technique was used for visualisation of IVR-free, cancer-specific and overall survival during follow up for IDM- vs. IDM+ as well as for the diagnostic subgroups A to D separately. Test for differences between curves were assessed by log rank test. Further comparison between groups were only performed for IDM- vs. IDM+ since too few patients remained in group B and C.

Hazard ratios for risk of IVR, death from urothelial cancer and all-causes comparing IDM- vs. IDM+ were estimated from date of surgery using multivariable Cox regression with adjustment for multiple confounders (age (categories), gender, clinical tumour stage, tumour location (renal pelvis/ureter/both), ipsilateral bladder cuff excision, previous bladder cancer, comorbidity and educational level). Start of follow up was date of surgery of UTUC and December 31th 2019 was regarded as the administrative end of follow-up. Patients were followed to date of IVR or were censored at date of death, emigration or end of follow-up whatever happened first.

Sensitivity analyses was performed by applying regression models after exclusion of the 287 patients with previous bladder cancer diagnosis, as well as in patients subjected to RNU, i.e. after excluding those operated with a segmental ureteric resection or nephrectomy only. Additionally, separate assessment of IVR for IDM- vs. IDM+ in ureteric- and renal pelvis cancer was motivated by detection of an association to tumour location in the previous multivariable analysis.

Lastly, for evaluation of potential influence on our results from heterogeneity in usage of IDM among the different hospitals, proportion of IDM+ was defined per hospital and visualized in a funnel-plot based on the number of patients per hospital including expected 95% variation. This putative association was also tested using binominal logistic regression with hospital size as a continuous variable based on number of included patients.

For all statistical analyses the R statistical package version 4.1.1 was used [14].

### Ethical statement

The study was approved by the Research Ethics Board of Uppsala University, Sweden (EPN 2015/277 and 2022-01747-02).

## Results

Baseline patient characteristics and treatment details are available in Table 1.

**Table 1:** Background characteristics and treatment details in patients with or without preoperative invasive diagnostic modalities (IDM).

		IDM- (n = 502)	%	IDM+ (n = 536)	%
**Age at surgery**	-64	65	13	82	15
	65–74	209	42	210	39
	75–80	129	26	151	28
	81-	99	20	93	17
**Gender**	Female	182	36	211	39
	Male	320	64	325	61
**Side**	Right	236	47	262	49
	Left	264	53	271	51
	Bilateral	2	0.4	3	0.6
**Previous bladder cancer**	Yes	138	27	149	28
	No	364	73	387	72
**Previous contralateral UTUC**	Yes	35	7.0	58	11
	No	467	93	478	89
**Education level**	Low	178	36	190	35
	Intermediate	199	40	190	35
	High	112	22	110	21
	Missing	13	2.6	6	1.1
**Comorbidity (CCI)**	0	204	41	204	38
	1	57	11	54	10
	2	138	27	146	27
	≥3	103	21	131	24
	Missing	0	0	1	0.2
**Clinical tumour stage**	Ta	189	38	247	46
	T1	75	15	79	15
	T2	60	12	47	8.8
	T3	126	25	90	17
	T4	18	3.6	16	3.0
	Tis	12	2.4	27	5.0
	Tx	18	3.6	28	5.2
	Missing	4	0.8	2	0.4
**Clinical nodal stage**	N0	392	78	388	72
	N1	16	3.2	18	3.4
	N2	14	2.8	20	3.7
	Nx	79	16	109	20
	Missing	1	0.2	1	0.2
**Type of surgery**					
*Nephroureterectomy*	Robotic	105	21	123	23
	Open	260	52	250	47
	Laparoscopic	44	8.8	75	14
*Segmental resection*	Robotic	7	1.4	3	0.6
	Open	51	10	59	11
	Laparoscopic	2	0.4	6	1.1
*Nephrectomy only*	Open	20	4.0	12	2.2
	Robotic	8	1.6	0	0
	Laparoscopic	6	1.2	7	1.3
**Bladder cuff excision**	Yes	246	49	299	56
	No	163	33	149	28
	Missing	93	19	88	16
**Systemic oncologic treatment**	No	468	93	499	93
	Neoadjuvant	10	2.0	7	1.3
	Induction	2	0.4	4	0.7
	Adjuvant	15	3.0	17	3.2
	Palliative	3	0.6	6	1.1
	Other	2	0.4	2	0.4
	Missing	2	0.4	1	0.2

IDM, invasive diagnostic modalities; CCI, Charlson Comorbidity Index.

Proportion of patients in groups based on preoperative diagnostic modalities and tumour location is presented in Table 2, showing similar distribution for ureter and renal pelvis tumour location.

**Table 2:** Proportion of patients in groups of invasive diagnostic modalities (IDM) based on tumour location.

	Renal pelvis	%	Ureter	%	Both	%	Total	%
**A**	306	50	183	46	13	35	502	48
**B**	40	6.6	24	6.0	8	22	72	6.9
**C**	60	9.9	19	4.8	3	8.1	82	7.9
**D**	200	33	169	43	13	35	382	37
**Total**	606		395		37		1038	

A) voided urine cytology, B) retrograde/antegrade pyelography and/or selective urine cytology, C) ureteropyeloscopy with or without barbotage for cytology, or D) ureteropyeloscopy with tumour biopsy.

**Fig 1:**
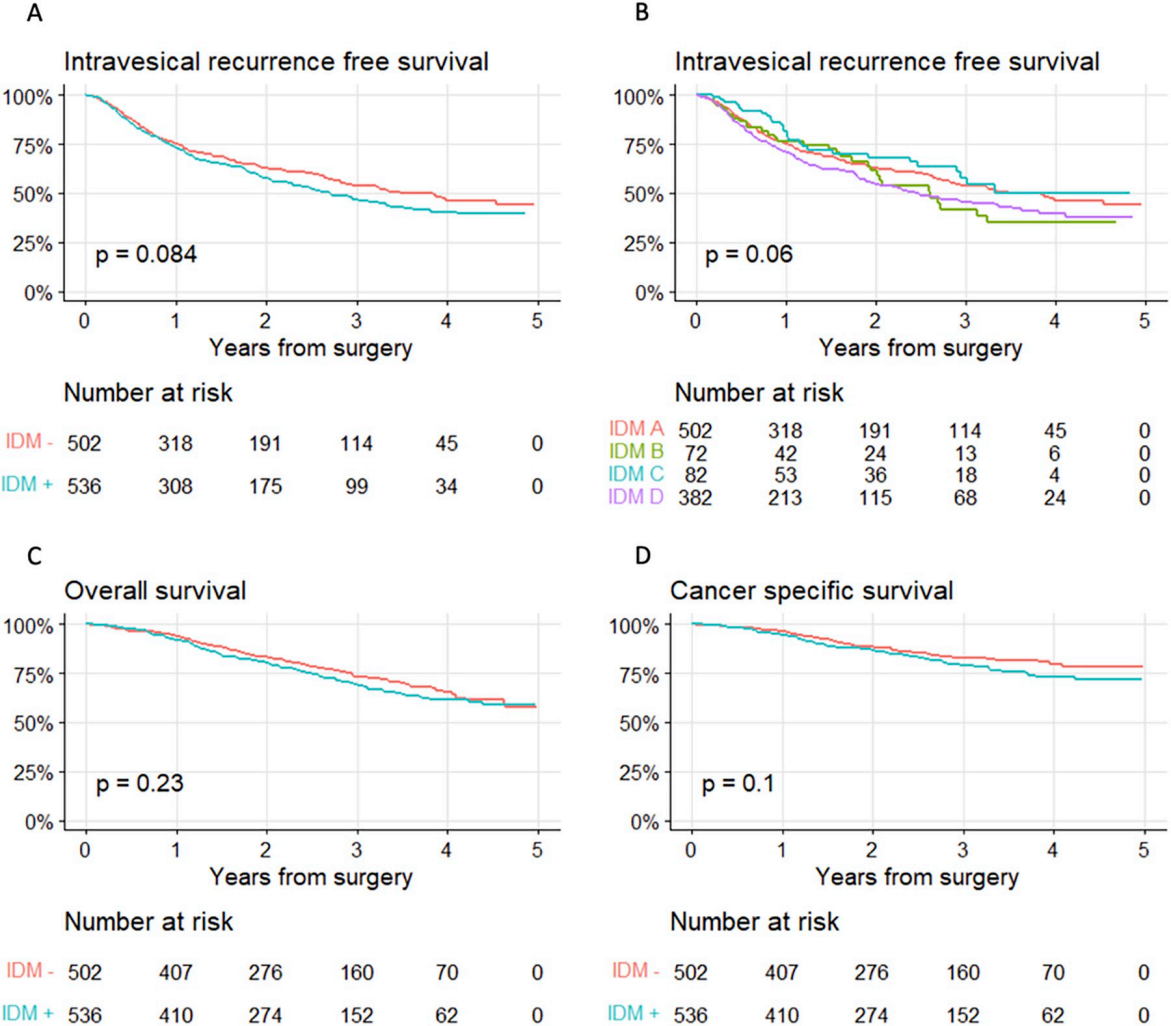
Kaplan-Meier graphs of survival for patients +/- preoperative invasive diagnostic modalities (IDM). a, intravesical recurrence-free survival; b, intravesical recurrence-free survival in diagnostic subgroups A) voided urine cytology, B) retrograde/antegrade pyelography and/or selective urine cytology, C) ureteropyeloscopy with or without obtaining barbotage cytology, or D) ureteropyeloscopy with tumour biopsy; c, overall survival; d, cancer-specific survival.

IVR was recorded in 120/536 and 100/502 patients respectively for those subjected to IDM or not after a median follow-up time of 1.3 (Inter Quartile Range (IQR) 0.6–2.6) yrs. IVR-free, cancer-specific and overall survival is visualised in Fig 1.

Using Cox multivariable regression models, the use of IDM was associated with IVR (HR 1.24, 95% CI 1.03–1.52) compared to no IDM. In similar multivariable analysis, the risk of cancer-specific death (HR 1.56, CI 1.12–2.18) differed between groups with worse outcome for patients subjected to preoperative IDM. All-cause death assessment showed HR 1.28 (CI 0.99–1.66) (Table 3).

**Table 3:** Multivariable Cox regression analysis of intravesical recurrence (IVR), cancer-specific death and all-cause death in groups of patients with or without invasive diagnostic modalities (IDM). Hazard ratios (HR) displayed for each outcome are adjusted for tumour location, sex, age, tumour stage, bladder cuff excision, previous bladder cancer, comorbidity (Charlson Comorbidity Index), and education level.

			IVR			Cancer-specific death			All-cause death		
	IDM	n	HR	95% CI	p	HR	95% CI	p	HR	95% CI	p
All	-	502	1	-	-	1	-	-	1	-	-
	+	536	1.24	1.02–1.52	0.031	1.56	1.12–2.18	0.008	1.28	0.99–1.66	0.057
Nephroureterectomy only	-	409	1	-	-	1	-	-	1	-	-
	+	448	1.18	0.94–1.47	0.147	1.75	1.20–2.56	0.004	1.37	1.03–1.83	0.032
Excluding previous bladder cancer	-	364	1	-	-	1	-	-	1	-	-
	+	387	1.39	1.08–1.78	0.011	1.64	1.09–2.48	0.019	1.23	0.90–1.67	0.189
Renal pelvis cancer	-	306	1	-	-	1	-	-	1	-	-
	+	300	1.07	0.81–1.41	0.624	1.27	0.82–1.97	0.279	1.14	0.81–1.60	0.446
Ureteric cancer	-	183	1	-	-	1	-	-	1	-	-
	+	212	1.66	1.21–2.28	0.002	2.19	1.22–3.93	0.009	1.59	1.03–2.47	0.037

HR, hazard ratio; IDM, invasive diagnostic modalities.

During follow-up of the total cohort of 1038 patients 250 (24%) patients died of any cause and 153 (15%) of urothelial cancer specifically. The corresponding proportions based on tumour location showed 149 (25%) and 91 (15%) deaths among 606 patients with tumours in the renal pelvis or 94 (24%) and 56 (14%) out of 395 patients with tumours in the ureter.

A sensitivity analysis excluding patients with previous bladder cancer diagnosis resulted in a similar IVR estimate (HR of 1.39, CI 1.08–1.78) for IDM+ vs IDM-. Separate assessment of IDM+ vs IDM- based on tumour location resulted in increased IVR in ureter tumours (HR 1.66, CI 1.21–2.28)) but not in renal pelvis tumours (HR 1.07, CI 0.81–1.41) (Table 3).

Evaluation of the potential effect of hospital size on the usage of IDM showed a small but statistically significant negative correlation (B = -0.005, p<0.001), with a higher proportion of IDM in smaller hospitals (S1 Fig).

## Discussion

This population-based study in all diagnosed Swedish UTUC patients 2015–2019 showed a 24% increased risk of IVR in patients subjected to any preoperative invasive diagnostic modalities (IDM) of the upper urinary tracts. Furthermore, IDM was associated with urothelial cancer death (HR 1.56, CI 1.12–2.18). Separate analyses stratified based on tumour location displayed an increased risk of IVR (66%) after IDM in tumours located in the ureter, while such risk increase could not be detected in the subset of patients with renal pelvic tumours only (HR 1.07, CI 0.81–1.41). Smaller hospital size was associated with a more frequent use of IDM.

Our present data is in line with findings in three recent meta-analyses and systemic reviews based on mainly small single-centre series, that points to higher occurrence of IVR after preoperative URS in patients treated with RNU [2, 4, 15]. However, a population-based study investigating IVR in 5713 Taiwanese patients with UTUC operated with RNU did not report a higher risk of IVR associated with diagnostic URS [6]. A comparison of outcomes in this population-based study and our results needs to consider demographic differences including different etiology of UTUC in Taiwan [16], but also the lack of adjustment of tumour-related confounders in the Taiwanese cohort. Also, the reported HR (1.14 (CI 1.0–1.3)) for IVR associated with preoperative URS actually do not exclude such an effect. Based on hypothesis-generating findings that also ureteric catheterisation might increase the risk of IVR [3] and a higher risk of IVR if URS is combined with a biopsy [17], we also performed stratified analyses based on the “severeness” of the exposure, from ureteral catheterization, ureteroscopy without biopsy to URS with biopsy (Fig 1B). However, related to few individuals in the intermediate risk groups, we merged all patients into one group (IDM+) during further analyses. Thus, in comparison to previous publications it is important to note that the exposed group in our data also include patients (6.9%) without URS (IDM group B, Table 2).

Our national registries allowed us to control for important patient-related factors such as comorbidity and socio-economic status, enabling assessment of overall- and cancer-specific survival. The adjusted analysis of cancer-specific death showed worse outcome for patients subjected to IDM, which is a novel finding and in contrast to previous studies [2, 18]. Possible explanations for the different survival outcomes in previous studies and the current are the population-based setting and inclusion of also segmental ureteric resections in addition to RNU in the current study. Furthermore, the association between use of IDM and hospital size in the current study and the reported association between hospital volume and outcome after RNU [19] could also have affected the survival analysis if speculated that low volume operating units subject patients to both higher IDM usage and mortality risk. Still, treatment delay due to prolonged diagnostic work-up associated with IDM [20], as well as hypothetical dissemination of tumour cells into periureteral soft tissue in conjunction with ureteroscopy [21] are putative explanations for decreased survival among patients in the IDM+ group. Consequently, it is reasonable to include all-cause and cancer-specific death in addition to IVR in future prospective evaluations of UTUC diagnostics.

The proportion of ureteric (38%) and renal pelvic (62%) cancer in the current study is similar to what has been reported previously [15]. Yet, we found a differential risk of IVR after IDM in patients with ureteric tumour location compared to tumours located in the renal pelvis only. To the authors knowledge, there is only one previous study reporting increased risk of IVR after IDM in patients with ureteric tumours compared to renal pelvis tumours [22]. In that study, where ureteric tumour location was further stratified in proximal, mid and distal tumours, proximal ureteric tumours were associated with increased risk of IVR, although with a broad confidence interval (HR 2.24, CI 1.00–5.04). However, as we did not have information on exact tumour location for patients with ureteric tumours, we were not able to test this possible association in the current study. Still, it could be speculated that instrumentation in the ureter leads to more direct physical contact with exophytic tumour tissue leading to more extensive detachment of tumour cells that subsequently might result in a higher rate of seeding to the bladder mucosa.

Previous bladder cancer is an established risk factor for IVR [15], which is also evident in our data. Yet exclusion of these patients did not change the main outcome.

Limitations of the current study is the non-random allocation to IDM which may introduce selection bias. However, we were able to control for a number of possible confounders using the detailed information on tumour stage and the linked data. Furthermore, there is a relatively short follow-up and we lack information on variant histology, tumour size or multifocality. Also, no information is available regarding specific technical aspects of the diagnostic procedures such as the use of an access sheath during URS which may impact IVR risk according to a recent report [23]. Smoking status, or information on preoperative hydronephrosis are also variables that are missing in our data, yet these may not imply increased risk of IVR [15]. In contrary, the referred meta-analysis by Seisen *et al*. reported other risk-factors for IVR that potentially could affect our results such as preoperative chronic kidney disease, positive urine cytology and tumour necrosis as well as treatment variables such as extravesical bladder cuff excision and positive surgical margins [15]. Furthermore, information on early adjuvant instillations is not available. However, to our knowledge the usage of such treatment has not yet been widely adopted in Sweden even if current guidelines recommend instillation of a single dose of intravesical chemotherapy in the early post-operative (2–10 days) period after RNU (and might be considered even after segmental resection) for UTUC [12]. There is however no recommendation regarding adjuvant treatment after URS or other instrumentation of the upper urinary tract since such data is still lacking. Another limitation derives from the registered low rate of complete bladder cuff excision (Table 1). This lack of adherence to an established quality indicator in UTUC extirpative surgery [24] may decrease the generalizability of the results.

Strengths of the current study are the population-based setting and linkage of data from national registries with high coverage enabling us to control for several important patient-related factors, as well as inclusion of catheterization of the upper urinary tract as exposure.

Likely, future development in diagnostic tools such as specific urine biomarkers as well as further development of oncological treatment regimens will lead to changes in diagnostic algorithms for UTUC. Based on both our present results and previous reports linking IVR risk to IDM, planning of such strategies should preferably include instrumentation of the upper tract in selected cases only as well as measures to an associated risk increase for these patients.

In conclusion, our findings suggest avoidance of unnecessary preoperative invasive diagnostic measures that might result in worse oncological outcomes in patients with UTUC, especially in patients with ureteric tumour location and/or advanced clinical tumour stage. Preferably, all UTUC patients should be discussed in a multidisciplinary tumour board (MDT) setting also involving experienced uro-radiologists and uro-pathologists [25]. Our finding of more frequent use of IDM in smaller hospitals also support such strategy. Future studies in patients where IDM is warranted should include stratification by tumour location and preferably also technical aspects of upper tract instrumentation and adjuvant bladder instillation treatment after diagnostic URS, as investigated in an ongoing randomized trial (clinicaltrials.org NCT02740426).

## Supporting information

S1 Fig Funnel plot of hospital size with proportion of patients subjected to invasive diagnostic modalities (IDM+). (DOCX)

S1 Table Distribution subjected to UTUC surgery per study year. Data from the Swedish National Registry of Urinary Bladder Cancer (SNRUBC).
(DOCX)

S2 Table Subgroups based on preoperative invasive diagnostic modalities (IDM). Distribution by hospital.
(DOCX)
