# Supplementary material for: Preoperative upper tract invasive diagnostic modalities are associated with intravesical recurrence following surgery for upper tract urothelial carcinoma: A population-based study
Source: PLoS One. 2023 Feb 2;18(2):e0281304. doi: 10.1371/journal.pone.0281304 (PMC9894449; doi:10.1371/journal.pone.0281304)

**S1 Fig.** Funnel plot of hospital size with proportion of patients subjected to invasive diagnostic modalities (IDM+)


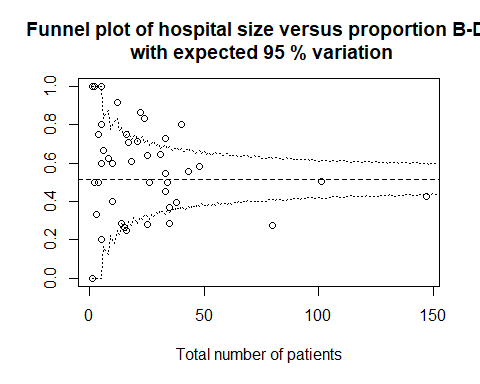

Supplement: S1 Fig — (DOCX) [file pone.0281304.s001.docx]
