# Supplementary material for: Preoperative upper tract invasive diagnostic modalities are associated with intravesical recurrence following surgery for upper tract urothelial carcinoma: A population-based study
Source: PLoS One. 2023 Feb 2;18(2):e0281304. doi: 10.1371/journal.pone.0281304 (PMC9894449; doi:10.1371/journal.pone.0281304)
Supplement: S1 Table — Data from the Swedish National Registry of Urinary Bladder Cancer (SNRUBC). (DOCX) [file pone.0281304.s002.docx]

**S1 Table.** Distribution subjected to UTUC surgery per study year. Data from the Swedish National Registry of Urinary Bladder Cancer (SNRUBC)

| Year | Kidney Pelvic Cancer | Ureteric cancer | Both | Total |
| --- | --- | --- | --- | --- |
| 2015 | 132 | 84 | 5 | 221 |
| 2016 | 150 | 73 | 5 | 228 |
| 2017 | 130 | 94 | 9 | 233 |
| 2018 | 109 | 84 | 12 | 205 |
| 2019 | 85 | 60 | 6 | 151 |
| Total | 606 | 395 | 37 | 1,038 |
