# Supplementary material for: Preoperative upper tract invasive diagnostic modalities are associated with intravesical recurrence following surgery for upper tract urothelial carcinoma: A population-based study
Source: PLoS One. 2023 Feb 2;18(2):e0281304. doi: 10.1371/journal.pone.0281304 (PMC9894449; doi:10.1371/journal.pone.0281304)
Supplement: S2 Table — Distribution by hospital. (DOCX) [file pone.0281304.s003.docx]

**S2 Table.** Subgroups based on preoperative invasive diagnostic modalities (IDM). Distribution by hospital.

| Hospital | A | B | C | D | B-D (+) | Total |
| --- | --- | --- | --- | --- | --- | --- |
| Total | 521 (48.3%) | 80 (7.4%) | 92 (8.5%) | 386 (35.8%) | 558 (51.7%) | 1079 |
| Malmö | 84 (57.1%) | 15 (10.2%) | 7 (4.8%) | 41 (27.9%) | 63 (42.9%) | 147 |
| Karolinska, Huddinge | 50 (49.5%) | 6 (5.9%) | 6 (5.9%) | 39 (38.6%) | 51 (50.5%) | 101 |
| Sahlgrenska | 58 (72.5%) | 6 (7.5%) | 3 (3.8%) | 13 (16.2%) | 22 (27.5%) | 80 |
| Umeå | 20 (41.7%) | 2 (4.2%) | 8 (16.7%) | 18 (37.5%) | 28 (58.3%) | 48 |
| Helsingborg | 19 (44.2%) | 18 (41.9%) | 2 (4.7%) | 4 (9.3%) | 24 (55.8%) | 43 |
| Danderyd | 8 (20.0%) | 1 (2.5%) | 4 (10.0%) | 27 (67.5%) | 32 (80.0%) | 40 |
| Uppsala, akad. | 23 (60.5%) | 1 (2.6%) | 0 (0.0%) | 14 (36.8%) | 15 (39.5%) | 38 |
| Södersjukhuset | 22 (62.9%) | 0 (0.0%) | 2 (5.7%) | 11 (31.4%) | 13 (37.1%) | 35 |
| Linköping | 25 (71.4%) | 4 (11.4%) | 3 (8.6%) | 3 (8.6%) | 10 (28.6%) | 35 |
| Uddevalla | 17 (50.0%) | 1 (2.9%) | 3 (8.8%) | 13 (38.2%) | 17 (50.0%) | 34 |
| Varberg | 18 (54.5%) | 1 (3.0%) | 4 (12.1%) | 10 (30.3%) | 15 (45.5%) | 33 |
| Jönköping | 15 (45.5%) | 4 (12.1%) | 3 (9.1%) | 11 (33.3%) | 18 (54.5%) | 33 |
| Örebro | 9 (27.3%) | 1 (3.0%) | 6 (18.2%) | 17 (51.5%) | 24 (72.7%) | 33 |
| Karlstad | 11 (35.5%) | 0 (0.0%) | 2 (6.5%) | 18 (58.1%) | 20 (64.5%) | 31 |
| Luleå | 13 (50.0%) | 2 (7.7%) | 4 (15.4%) | 7 (26.9%) | 13 (50.0%) | 26 |
| Falun | 18 (72.0%) | 0 (0.0%) | 0 (0.0%) | 7 (28.0%) | 7 (28.0%) | 25 |
| Sundsvall | 9 (36.0%) | 4 (16.0%) | 3 (12.0%) | 9 (36.0%) | 16 (64.0%) | 25 |
| Borås | 4 (16.7%) | 1 (4.2%) | 4 (16.7%) | 15 (62.5%) | 20 (83.3%) | 24 |
| Skövde | 3 (13.6%) | 0 (0.0%) | 3 (13.6%) | 16 (72.7%) | 19 (86.4%) | 22 |
| Sankt Göran | 6 (28.6%) | 2 (9.5%) | 3 (14.3%) | 10 (47.6%) | 15 (71.4%) | 21 |
| Eskilstuna | 7 (38.9%) | 0 (0.0%) | 1 (5.6%) | 10 (55.6%) | 11 (61.1%) | 18 |
| Norrköping | 5 (29.4%) | 1 (5.9%) | 3 (17.6%) | 8 (47.1%) | 12 (70.6%) | 17 |
| Karlskrona | 12 (75.0%) | 1 (6.2%) | 0 (0.0%) | 3 (18.8%) | 4 (25.0%) | 16 |
| Västerås | 4 (25.0%) | 2 (12.5%) | 3 (18.8%) | 7 (43.8%) | 12 (75.0%) | 16 |
| Gävle-Sandviken | 11 (73.3%) | 0 (0.0%) | 0 (0.0%) | 4 (26.7%) | 4 (26.7%) | 15 |
| Kalmar | 10 (71.4%) | 0 (0.0%) | 1 (7.1%) | 3 (21.4%) | 4 (28.6%) | 14 |
| Kristianstad | 1 (8.3%) | 1 (8.3%) | 3 (25.0%) | 7 (58.3%) | 11 (91.7%) | 12 |
| Östersund | 4 (40.0%) | 1 (10.0%) | 0 (0.0%) | 5 (50.0%) | 6 (60.0%) | 10 |
| Växjö | 4 (40.0%) | 0 (0.0%) | 2 (20.0%) | 4 (40.0%) | 6 (60.0%) | 10 |
| Ljungby | 6 (60.0%) | 0 (0.0%) | 3 (30.0%) | 1 (10.0%) | 4 (40.0%) | 10 |
| Halmstad | 3 (37.5%) | 0 (0.0%) | 0 (0.0%) | 5 (62.5%) | 5 (62.5%) | 8 |
| Eksjö-Nässjö | 2 (33.3%) | 2 (33.3%) | 1 (16.7%) | 1 (16.7%) | 4 (66.7%) | 6 |
| Västervik | 2 (33.3%) | 0 (0.0%) | 0 (0.0%) | 4 (66.7%) | 4 (66.7%) | 6 |
| Hudiksvall | 2 (40.0%) | 1 (20.0%) | 2 (40.0%) | 0 (0.0%) | 3 (60.0%) | 5 |
| Värnamo | 0 (0.0%) | 0 (0.0%) | 1 (20.0%) | 4 (80.0%) | 5 (100.0%) | 5 |
| Ängelholm | 1 (20.0%) | 1 (20.0%) | 0 (0.0%) | 3 (60.0%) | 4 (80.0%) | 5 |
| Södertälje | 1 (25.0%) | 0 (0.0%) | 0 (0.0%) | 3 (75.0%) | 3 (75.0%) | 4 |
| Nyköping | 2 (50.0%) | 0 (0.0%) | 0 (0.0%) | 2 (50.0%) | 2 (50.0%) | 4 |
| Kungälv | 1 (25.0%) | 0 (0.0%) | 1 (25.0%) | 2 (50.0%) | 3 (75.0%) | 4 |
| Bollnäs | 2 (66.7%) | 0 (0.0%) | 0 (0.0%) | 1 (33.3%) | 1 (33.3%) | 3 |
| Ystad | 0 (0.0%) | 1 (50.0%) | 0 (0.0%) | 1 (50.0%) | 2 (100.0%) | 2 |
| Karolinska, Solna | 1 (50.0%) | 0 (0.0%) | 0 (0.0%) | 1 (50.0%) | 1 (50.0%) | 2 |
| Lundby | 0 (0.0%) | 0 (0.0%) | 1 (50.0%) | 1 (50.0%) | 2 (100.0%) | 2 |
| Alingsås | 1 (50.0%) | 0 (0.0%) | 0 (0.0%) | 1 (50.0%) | 1 (50.0%) | 2 |
| Mora | 1 (100.0%) | 0 (0.0%) | 0 (0.0%) | 0 (0.0%) | 0 (0.0%) | 1 |
| Visby | 0 (0.0%) | 0 (0.0%) | 0 (0.0%) | 1 (100.0%) | 1 (100.0%) | 1 |
| Katrineholm | 1 (100.0%) | 0 (0.0%) | 0 (0.0%) | 0 (0.0%) | 0 (0.0%) | 1 |
| Örnsköldsvik | 1 (100.0%) | 0 (0.0%) | 0 (0.0%) | 0 (0.0%) | 0 (0.0%) | 1 |
|  | 4 (80.0%) | 0 (0.0%) | 0 (0.0%) | 1 (20.0%) | 1 (20.0%) | 5 |
